# Integrated Dynamic Photovoltaic Facade for Enhanced Building Comfort and Energy Efficiency

**DOI:** 10.3390/biomimetics9080463

**Published:** 2024-07-31

**Authors:** Masoud Valinejadshoubi, Andreas K. Athienitis, Ashutosh Bagchi, Matin Abtahi

**Affiliations:** Centre for Zero Energy Building Studies, Department of Building, Civil, and Environmental Engineering, Concordia University, Montreal, QC H3G 1M8, Canada; andreask.athienitis@concordia.ca (A.K.A.); ashutosh.bagchi@concordia.ca (A.B.); matinabtahi@gmail.com (M.A.)

**Keywords:** dynamic facades, BIPV/T, control systems, daylight factor, energy

## Abstract

This simulation study explores the potential of a novel façade design with integrated control system comprising a dynamic photovoltaic (PV) facade integrated with dimming lighting control to enhance the work environment in office buildings and achieve energy-efficient solutions. Parametric modeling using the Grasshopper plug-in for Rhino software 7, coupled with energy simulation through the Honeybee environmental plug-in for the EnergyPlus program, are used in the methodology. The integrated control strategy was simulated to study in a single office space, utilizing the Daysim engine to assess indoor daylight quality and focusing on Daylight Factor (DF) and Daylight Glare Probability (DGP). Additionally, two artificial lighting control systems were examined for potential integration with the dynamic PV facade to minimize lighting load. The study employs the Galapagos evolutionary solver function embedded within Grasshopper to identify optimum solutions. The dynamic PV façade achieves substantial reductions in overall energy consumption, cutting it by 73% in June, 54% in July, 54.5% in August, and 52.55% in September. The results demonstrate substantial reductions in total energy consumption, with notable savings in heating and cooling due to the dynamic facade’s ability to balance and control solar radiation during working hours. Moreover, the dynamic PV facade contributes to electricity generation, demonstrating its potential to improve visual comfort, decrease energy consumption, and generate electric energy through rotational adjustments and varying transparency levels.

## 1. Introduction

A substantial portion of global energy consumption and greenhouse gas emissions arises from buildings [1], surpassing even the energy usage in industry and transportation, constituting nearly one-third of the world’s energy demand [2]. Among the various components of a building, electrical lighting, heating, and cooling contribute most to overall energy consumption. According to Waide and Tanishima [3], as reported by the International Energy Agency (IEA), grid-based electric lighting globally consumes 19% of total electricity production, with lighting representing an average of 34% of electricity consumption in the tertiary sector [4,5].

**Figure 1:**
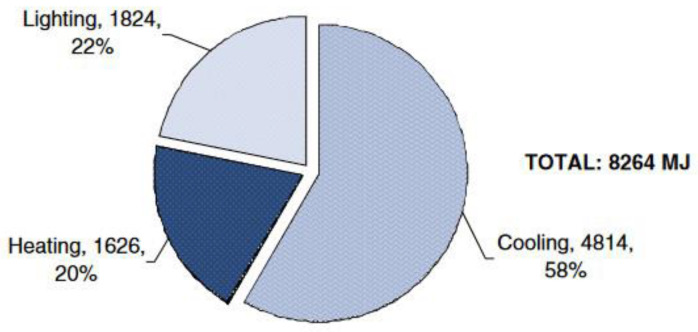
Energy consumption (MJ) for lighting, heating and cooling of a highrise office building in Montreal [6].

Figure 1 shows that lighting accounts for 22% of energy consumption of a typical highrise office building in Montreal, while space heating and cooling contribute 20% and 58%, respectively. By effectively utilizing and controlling daylight in office buildings, there is a significant potential for reducing energy consumption related to lighting, cooling in the summer, and heating in the winter. Additionally, this approach can enhance productivity among office workers [7]. However, buildings with extensive fenestration may experience fluctuating heating and cooling loads due to excessive solar gains, leading to glare issues, particularly on south-facing facades of office buildings. As a response, researchers and manufacturers are intensifying efforts toward designing energy-efficient buildings, incorporating renewable resources and passive design techniques to enhance energy efficiency and reduce operational energy demand [8,9,10,11]. Building Automation Systems (BASs) and Building Energy Management Systems (BEMSs) with enhanced environmental information offer exciting prospects to reduce building energy consumption and enhance comfort spaces. Given the significant influence of lighting, heating, and cooling on building energy consumption, particularly in office buildings, a holistic approach is necessary. This involves reducing energy demand, addressing lighting and thermal comfort, and deploying local efficient energy systems to meet additional demands [12]. The selection of energy and shading systems and control strategies, as highlighted by various sources [5,7], plays a crucial role in optimizing lighting and thermal performance in perimeter spaces.

In this context, dynamic building envelope technologies have garnered considerable attention due to their potential to enhance facade design, environmental responsiveness, and overall building energy performance, aiming to decrease energy demands and create comfortable office spaces [5,13,14]. Dynamic building envelopes must fulfill multiple requirements, such as light amount, uniformity, glare reduction, thermal comfort, and energy conservation. The concept introduces possibilities for creating a superior work environment in office buildings with energy-efficient solutions. Beyond controlling sunlight for heating, cooling, and glare, innovative facade systems may increase daylight levels and enhance uniformity within office spaces [15]. Dynamic facade concepts emerge as an effective strategy for managing outdoor and indoor interactions, with Building Integrated Photovoltaic (BIPV) systems and Semi-Transparent Photovoltaic (STPV) serving as integral components to bridge the gap between multifunctional dynamic facades and overall building energy balance. While BIPV and STPV are increasingly adopted as efficient solutions by designers and engineers [16,17], there remains a gap in research on optimizing such systems, considering factors such as Daylight Factor (DF), Daylight Glare Probability (DGP), artificial lighting control systems, heating, and cooling. 

In this paper, DF and DGP are considered as the main indices to assess the visual comfort in a south-faced office unit, which is explained in the following:

i. Daylight Factor (DF): The Daylight Factor is an important index for evaluating the adequacy of natural light in an interior space, providing a measure of the natural light available compared to the light available outdoors under overcast sky conditions. According to CIBSE [18], Daylight Factor is defined as the ratio of indoor illuminance at a specific point to the simultaneous outdoor illuminance, expressed as a percentage. This metric offers a simple yet powerful tool to gauge the natural lighting levels within a building.

Yose Rizala [19] stated that the Daylight Factor can be calculated using the following formula:

(1) $$DF=\left(\frac{E_{I}}{E_{O}}\right)\times 100\%$$

where *E_I_* is daylight illumination at a point on the workplane in the room, and *E_O_* is simultaneous outdoor illuminance on the horizontal. 

The Daylight Glare Index (DGI) is a principal metric for assessing discomfort glare from daylight, particularly when dealing with sources that have non-uniform luminance levels. Despite its importance, it does have some limitations and can be challenging to apply accurately. As defined by Hafiz [20], glare is the difficulty in seeing in the presence of bright light due to a direct or reflected light source within the visual field. This is typically quantified by comparing the size, location, and luminance of glare sources to the average luminance in the field of view, excluding the glare source itself.

Kurnia et al. [21] discussed that many studies use the concept of a glare index to measure indoor glare. A glare index is a quantitative measure indicating the level of perceived glare within a field of view. The general concept of the glare index can be expressed through a specific equation [21]:

(2) $$\mathrm{Glare}=\overset{n}{\underset{i=1}{\sum }}\frac{L_{s,i\;}^{ℱ}\omega_{s,i}}{L_{b}^{g}\;P_{i}^{h}}$$

While this equation provides a foundational concept, further research is necessary to refine it based on empirical data to determine an accurate glare index formula. Various experiments have led to the development of several glare indices, particularly for light sources such as daylight. In 1972, Hopkinson introduced the Daylight Glare Index (DGI), which accounts for the potential for significant glare sources, especially from the diffuse sky visible through a window.

The DGI remains a crucial index for evaluating daylight glare, especially from non-uniform luminance sources. However, its calculation is complex due to the need to determine geometric parameters, such as the solid angle and position index, and the luminance values perceived by the observer [22]. According to Hopkinson [23], the DGI formula is based on subjective perceptions. His research indicates that a DGI greater than 31 signifies intolerable glare, while a DGI less than 18 suggests that glare is barely perceptible. The DGI equation is as follows [23]:

(3) $$DGI=10\times log_{10}0.48\overset{n}{\underset{i=1}{\sum }}\frac{L_{s,i\;\;}^{1.6}\omega_{s,i}^{0.8}}{L_{b}+\left(0.07\omega_{s,i}^{0.5}\;L_{s,i}\right)}$$

### 1.1. Adaptive Facade Systems

The widespread adoption of glazed exterior facades in contemporary office buildings has revolutionized workplace design, offering a plethora of physical and psychological health benefits to occupants. However, the deployment of these transparent structures has brought about challenges related to thermal resistance and visual discomfort [16]. In response to these challenges, there has been a concerted effort in recent years to develop energy-conscious buildings, with a particular focus on advanced adaptive facade systems [24]. Just as natural organisms exhibit adaptive behavior to survive and thrive in their habitats, adaptive facade systems can dynamically adjust their properties based on external factors such as sunlight intensity, temperature, and wind. By incorporating sensors and actuators, these systems can mimic the responsiveness of natural organisms, optimizing energy performance and occupant comfort. Among these innovations, dynamic PV facades have emerged as a promising solution capable of dynamically responding to climatic variations, thereby enhancing both visual comfort and energy performance [25]. In Canada, where lighting, heating, and cooling constitute the highest portions of electrical energy consumption in office buildings, ample opportunities exist for research [26].

Studies by Lee and Selkowitz [26] on adoptable shading devices and auto-dimming lighting systems revealed significant reductions in cooling and lighting energy consumption, showcasing the potential of dynamic shading systems. Athienitis and Tzempelikos [15] demonstrated 75% energy savings on overcast days and 90% on clear days through an advanced window system with controlled shading and light dimming. Additionally, research by Zhou et al. [27] emphasized the energy-saving benefits of auto-dimming lighting, reporting a 57% reduction in lighting energy consumption compared to non-dimming lighting [28].

### 1.2. Optimizing Visual Comfort and Energy Efficiency

In office environments, achieving optimal visual comfort is essential for productivity and well-being. DF is a metric used to quantify the amount of natural light available in a room relative to the illumination on a horizontal plane under an unobstructed sky hemisphere [29,30]. It provides insight into the distribution of daylight within a space, helping designers ensure adequate lighting levels without causing glare or discomfort. A high DF indicates a well-lit room with ample natural light, reducing the need for artificial lighting and consequently lowering energy consumption.

On the other hand, DGP assesses the likelihood of glare occurring in space due to excessive brightness contrasts between light and dark areas [29,30]. Glare can cause discomfort, eye strain, and reduced visual performance, negatively impacting occupant satisfaction and productivity. By analyzing DGP, designers can identify potential glare sources and implement mitigation strategies to enhance visual comfort.

In the context of office units, where sunlight exposure is more intense, balancing DF and DGP becomes paramount. Biomimetic-inspired STPV windows offer a unique opportunity to address this challenge by mimicking nature’s adaptive strategies. Exploring STPV with varying transparency levels, Miyazaki et al. [31] found that optimum Semi-Transparent Photovoltaic (STPV) windows reduced total building electricity consumption by 55%, enhancing visual comfort and energy efficiency [32]. Drawing inspiration from natural systems, such as the self-regulating mechanisms found in plant leaves or animal eyes, designers can develop STPV windows and facades that dynamically adjust transparency levels based on incoming sunlight intensity. This biomimetic approach allows for optimized daylighting while minimizing glare, creating a harmonious and comfortable indoor environment reminiscent of natural ecosystems.

Despite the growing popularity of glazed exterior facades in modern office buildings due to their numerous benefits, including physical and psychological well-being, these structures present significant challenges. Issues related to thermal resistance and visual discomfort persist, prompting the need for innovative solutions. While extensive research has focused on developing energy-conscious buildings, particularly through adaptive facade systems, the integration of dynamic PV facades remains relatively unexplored. Furthermore, in regions like Canada, where lighting, heating, and cooling contribute significantly to electrical energy consumption in office buildings, a pressing need exists for sustainable and efficient building design solutions. Current studies highlight the potential of dynamic PV shading devices and auto-dimming lighting systems in reducing energy consumption, emphasizing the importance of implementing such technologies in building design. However, gaps remain in the literature regarding the integration of these systems and their impact on visual comfort and energy efficiency. This study aims to optimize visual comfort, reduce glare, and minimize energy consumption in glazed office buildings. By exploring the interplay between dynamic PV facades and lighting control systems, the research seeks to enhance the overall working environment and productivity while contributing to more efficient and sustainable building operations. Ultimately, this study will advance sustainable building design practices by providing insights and practical applications of adaptive building control systems, promoting the development of innovative and environmentally friendly building technologies. The usefulness of dynamic PV facades in this paper, inspired by biomimetics, lies in their ability to enhance sustainability and efficiency in building operations. By mimicking natural systems that adapt to environmental changes, these facades optimize visual comfort, reduce glare, and minimize energy consumption in glazed office buildings. They generate electricity, reduce the need for artificial lighting, and improve occupant comfort, thereby promoting innovative and sustainable building design practices rooted in the principles of biomimetics.

## 2. Research Methodology

The methodology section outlines the research approach, detailing the development of the dynamic PV facade system concept and its integration with artificial lighting control systems. The methodology encompasses several key components, starting with the conceptualization and parametric modeling of the dynamic PV facade system using Rhino/Grasshopper software 7. Rhino/Grasshopper, a graphical algorithm editor, enables rapid generation of parametric forms, bridging the gap between design concepts and energy performance considerations [33]. This computational tool facilitates the incorporation of biomimetic principles and adaptive behavior into the design process, allowing for the exploration of various facade configurations and lighting control strategies. The Rhino parametric software 7 integrates these design elements as multi-variable inputs in an optimization process aimed at achieving optimal outcomes, including high-quality daylight, minimal glare, and efficient electric energy consumption and generation [34]. Environmental simulations, encompassing visual comfort, electric lighting, heating, cooling, and internal energy analysis, are conducted using the Honeybee plug-in [35]. Integrated with Radiance and Daysim, Honeybee enables advanced grid-based daylight simulation and calculation of illuminance [9] and DGP through image-based simulation to assess the performance of the dynamic PV facade system.

### 2.1. Overall Concept and Design

Biomimetics, or biomimicry, is an interdisciplinary approach that draws inspiration from nature to solve human challenges and innovate in various fields, including architecture and technology. By studying biological systems, organisms, and ecosystems, biomimetics seeks to emulate their efficient structures, behaviors, and processes to design more sustainable, functional, and adaptive solutions. In the context of our research on dynamic PV facade systems, biomimetics informs the circular shape of the STPV and PV layers, mirroring natural forms observed in organisms. Additionally, biomimetic principles guide the system’s adaptive function, reflected in the independent movement of PV layers to optimize solar energy capture and regulate daylight levels, akin to adaptive behaviors found in nature. The primary objective of this study is to create a dynamic facade solution capable of regulating daylight, reducing glare, and optimizing solar energy capture in office environments. By integrating biomimetic principles and advanced control systems, our dynamic PV facade model aims to balance natural lighting, energy consumption, and occupant comfort harmoniously.

**Figure 2:**
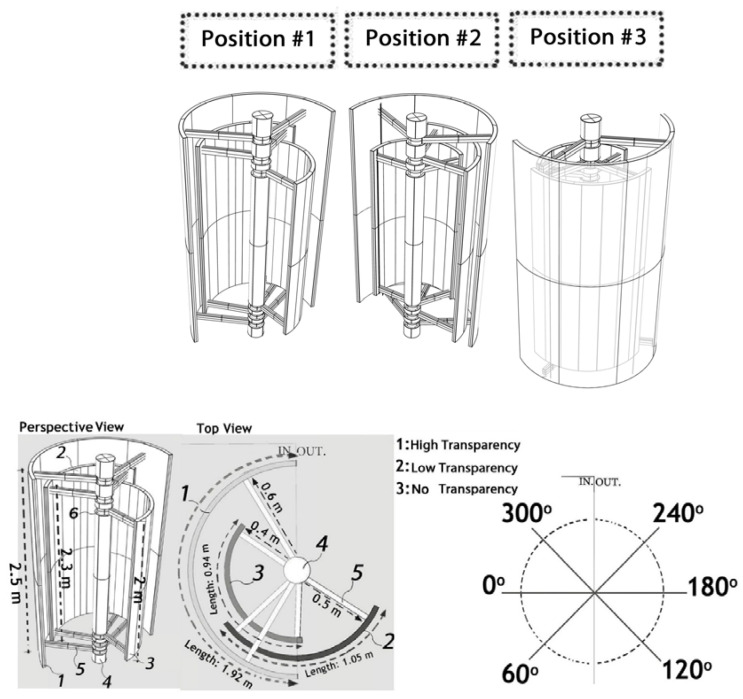
**Top**: The example of three positions of the dynamic PV system; **Bottom Right**: the diagram depicts the rotation degrees of the dynamic PV system consisting of layers 1, 2, 3, demonstrating the range of motion. **Left**: a detailed perspective and top view illustrate the components and structure of a module within the dynamic PV system.

**Figure 3:**
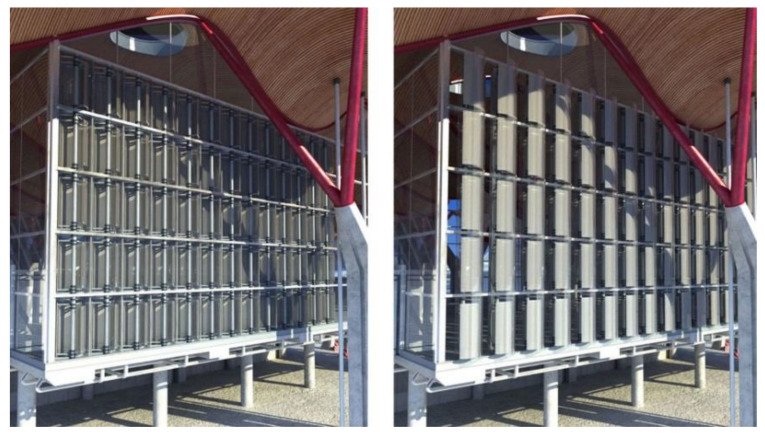
Conceptual rendering of the dynamic PV facade on an office building.

The dynamic system comprises two Semi-Transparent Photovoltaic (STPV) layers, thin-film amorphous silicon glass with varying levels of transparency (Figure 2, No. 1 and 2), and one Polycrystalline PV layer (Figure 2, No. 3). Each PV layer possesses an independent structure (No. 5) connected to the central structural part (No. 4), allowing individual movement through dedicated centric servomotors (No. 6). A servomotor, serving as a rotary actuator, facilitates precise control over angular position, velocity, and acceleration [36]. The three PV layers, along with their structural and electrical components, collectively form a modular device. The central structural part is designed to connect to either the primary or secondary building beam, enabling the transfer of the system’s load (No. 4). Figure 2 illustrates the simulated dimensions of one module of dynamic PV device in this research, and Figure 3 illustrates conceptual rendering of the dynamic PV facade on an office building. The rotations of the PV layers were specified in six steps (0°, 60°, 120°, 180°, 240°, 300°), as illustrated in Figure 2. In addition to the material properties of the PV layers within the dynamic system (Table 1), the materials used for the interior surfaces of the building (Table 1 and Table 2) are paramount for controlling daylight and glare, thereby influencing visual comfort or discomfort.

The main design considerations for the dynamic PV facade system include:

- Biomimetic Design: Drawing natural inspiration to optimize system operation, structures, and behaviors.
- Modular Structure: Each PV layer has an independent structure, allowing for individual movement.
- Precise Control: Utilizing servomotors for accurate adjustment of PV layer positions.
- Material Selection: Choosing appropriate materials to balance energy generation and visual comfort.
- Rotational Adjustments: Specifying rotations to optimize solar energy capture throughout the day.

### 2.2. Lighting Control System Parameters

The dynamic PV facade functions as an automated system designed to regulate daylight within the necessary interior illuminance range (500 Lux) during office hours from 8 A.M. to 4 P.M. However, the DF provides an average lux value over broad surfaces, such as an entire room, which may not ensure uniform lighting throughout the office space [37]. Hence, artificial lighting becomes essential to achieve an appropriate lighting level in specific areas of the room. The artificial lighting control system is integrated with the dynamic PV facade to adopt the most effective hourly control strategy for reducing the lighting load.

**Figure 4:**
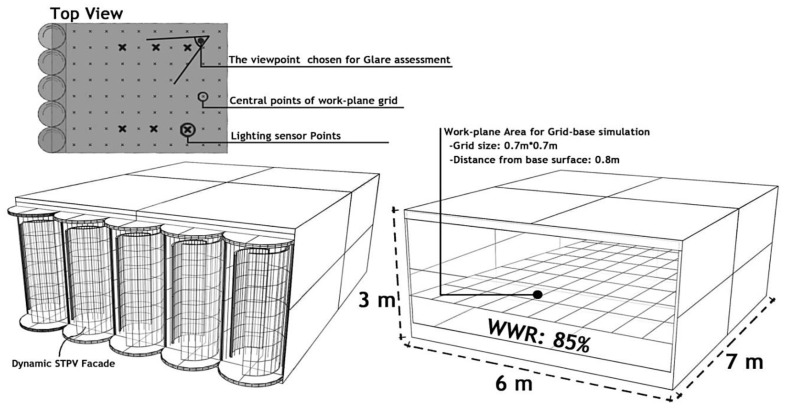
**Right**: The baseline office room model; **Left**: The office room model with dynamic PV facade.

Two artificial lighting control systems simulated in this research include (1) an Automated Switch-Off Occupancy Sensor and (2) an Always-On system during active occupancy working hours with auto-dimming. Following a lighting calculation, in simulation, six light sensors were placed in the further areas from the facade, receiving less daylight than the illuminance threshold (refer to Figure 4). These lighting sensors were simulated on the working table at a 1.40 m distance from each other, at a distance of 0.8 m from the floor, and 2.40 m from the facade. The lighting source chosen for this study was a light-emitting diode (LED) screw-base lamp with a power rating of 250 watts.

### 2.3. Energy Analysis

In this research study, a simulation-based approach was employed to analyze the energy performance of a dynamic PV facade system implemented within an office space situated in Montreal, ASHRAE climate zone 6, characterized as Cold–Humid. The simulation focused on three key components: building material, operational schedule, and HVAC system. Table 1 details the properties of the PV layers, while Table 2 outlines the recommended wall, roof, and floor materials by ASHRAE for the specified climate zone, along with Table 3 interior surface characteristics relevant for lighting analysis. This simulation-based methodology comprehensively evaluated the dynamic PV facade system’s energy performance under various environmental conditions and operational settings.

**Table 1:** Characteristics of PV layers in each analyzed module.

Element	Type	Max. Power (Wp/sqm) [38]	Visible Light Transmittance (%) [39]	SHGC (%) [39]	UV Transmittance (%) [39]	Exterior Reflection (%) [39]	Surface Area (Square Meter)
PV Layer 1	High Transparency	28	28	40	4	8.2	20
PV Layer 2	Low Transparency	40	10	29	0.1	8.3	10
PV Layer 3	Polycrystalline	200	0	0.5	0	0.7	8

**Table 2:** Characteristics of construction materials used in simulation.

Construction	Material (From Out to In)	U-Value	R-Value
Wall [7]	2.54 cm Stucco; 20.32 Concrete; Insulation; 1.27 cm Gypsum	0.425	2.349
Floor [7]	1.27 cm Gypsum; AtticFloor NonRes Insulation; 1.27 cm Gypsum	0.157	6.334
Roof [7]	Roof membrane; Roof insulation; Metal decking	0.18	5.36

**Table 3:** Characteristics of interior surfaces in simulation.

Interior Surfaces	Color	Roughness [40]	Secularity [40]
Interior Walls	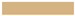	0.03	0
Interior Ceiling	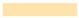	0.03	0
Interior Floor	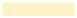	0.01	0.05

To estimate electricity generation, we first evaluated the hourly average global solar radiation incident on the PV layers (Q) in kWh/m² using the Honeybee plug-in. The transmittance of glass (ƮG), as defined in Table 1, played a critical role in this calculation. The efficiency or yield of the PV panels (η) in this study varied depending on the PV type, also detailed in Table 1.

The performance ratio (PR), which is used to assess the system’s overall performance, was established at 0.75, as referenced in previous studies [41,42]. With the radiation data in hand, we calculated the energy generation of the PV layers using the following equation [43]:

(4) $$Electricity\;Generation=PV_{\eta_{e}}\;\tau_{G}\;A_{PV}\;Q$$

This approach allowed us to precisely estimate the energy output of each PV layer by taking into account the specific characteristics of the glass transmittance, PV panel efficiency, and the established performance ratio.

**Figure 5:**
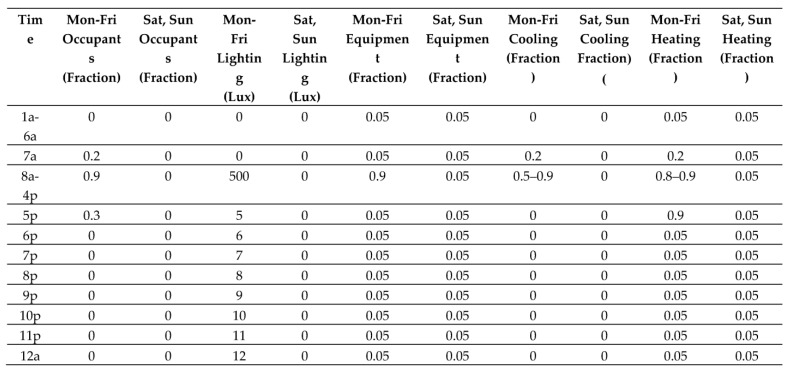
The schedule of the office room investigated in this research.

Figure 5 provides a comprehensive schedule for the office, encompassing occupants, lighting, equipment, and cooling and heating systems. Schedule input values are presented in fractional units within the range of 0 and 1, except for lighting, which is specified in lux. The regular workweek in Montreal spans 40 h, resulting in the office room being occupied from 8 A.M. to 4 P.M., with a density of 0.14 people/m² (six people for 42 m^2^). The standard lighting level for office work is set at 500 lux, representing the sensor setpoint for lights during working hours [29]. Equipment usage was assumed to be 8 W/m^2^, with some equipment operating continuously during operational hours.

Cooling and heating are assumed to be provided by Packaged Terminal Heat Pumps (PTHPs) with a coefficient of performance (COP) of 3.50. Night-time building setbacks were implemented (26 °C for cooling and 18 °C for heating) to reduce the temperature difference between ambient and indoor spaces, aiming to save energy. The cooling and heating systems were initiated at 5 AM, using 20% of the system capacity, to gradually balance the indoor air temperature within the setpoint range (24 °C for cooling and 20 °C for heating). Additionally, 5% of the heating system capacity was utilized to maintain an acceptable temperature during unoccupied periods, preventing equipment freezing in the office room in winter or overheating in summer. 

### 2.4. Simulation Setup

**Figure 6:**
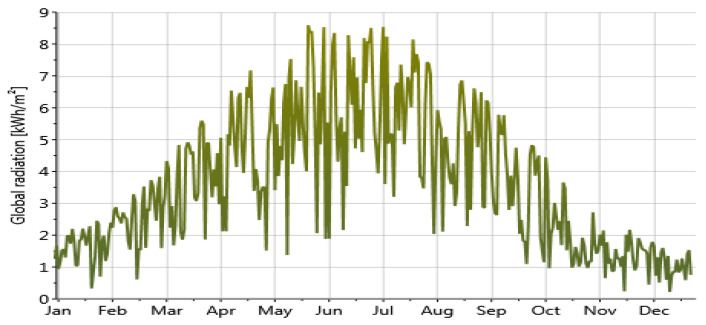
Daily global radiation in Montreal (Meteonorm 7 software).

**Figure 7:**
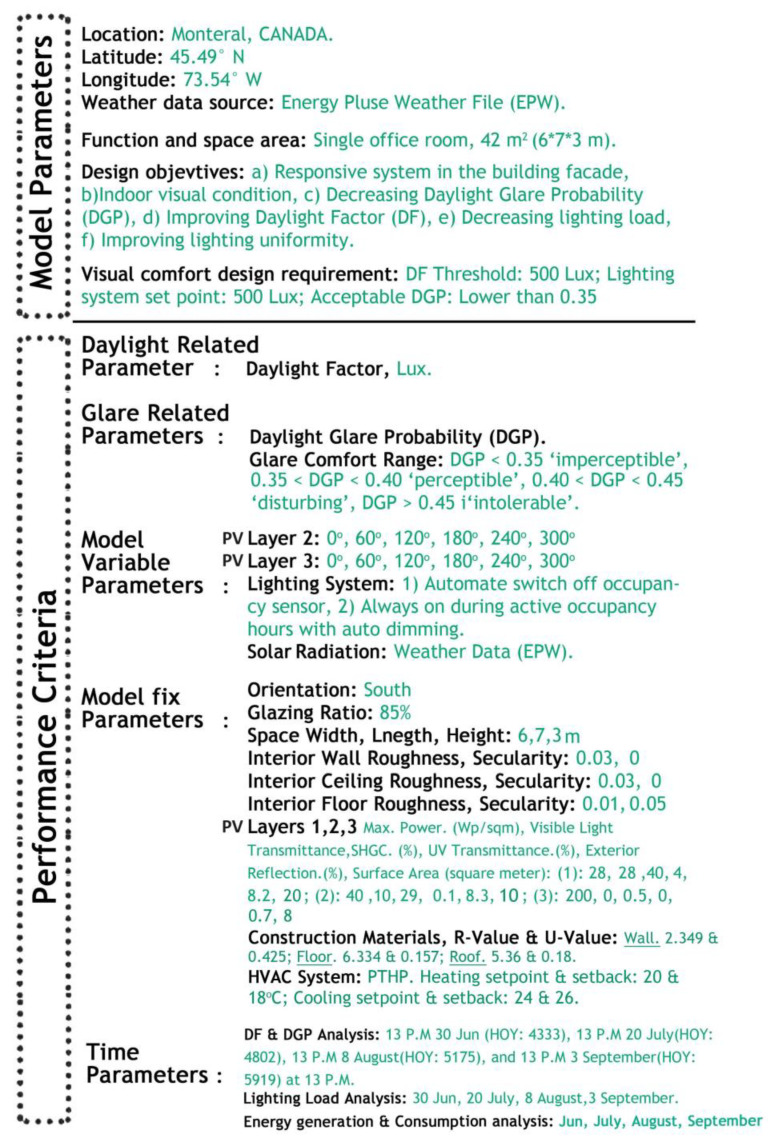
Model parameters and performance criteria in the simulation.

The methodology is demonstrated by considering a south-facing single office space located in Montreal, Canada. Solar radiation, a non-controllable input, plays a significant role in the evaluation process. Figure 6 illustrates the annual variation in global solar radiation in Montreal, with peak values occurring in June, July, August, and September. Figure 7 illustrates the model parameters and performance criteria utilized in the evaluation process. The primary variable parameters included PV layer rotation, two lighting control systems, and four analysis periods during the year integrated into the Honeybee/Grasshopper algorithmic process. 

**Figure 8:**
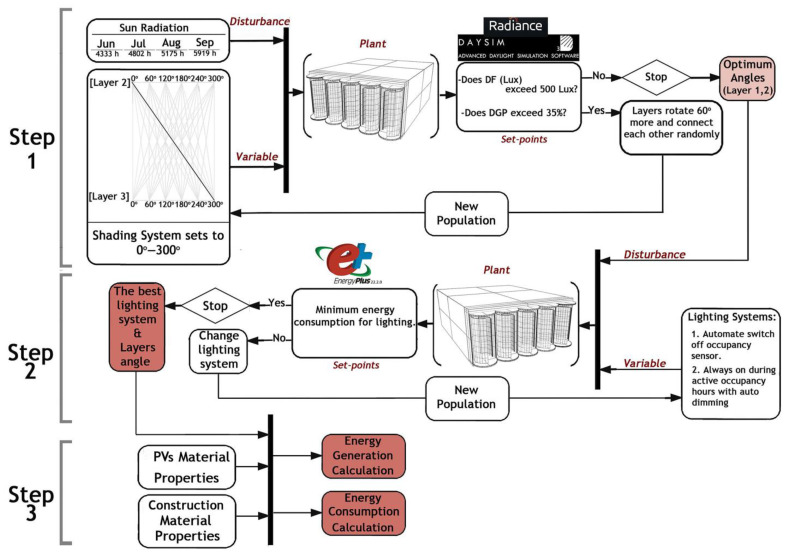
A visual representation of the constant feedback loop during the optimization processes.

Figure 8 provides a visual representation of the constant feedback loop during the exploration of control system scenarios, incorporating thresholds for illuminance, glare index, and lighting load. This exploration utilized a problem-solving exhaustive method through Galapagos, an evolutionary solver in Grasshopper known as a Genetic Algorithm (GA) [39]. Galapagos, functioning as a genetic multi-objective algorithm solver, operates based on a typical input–output relationship.

The optimization process involved defining three main objectives: achieving the required daylight illuminance threshold of 500 lux, reducing glaring risks in a specific occupant position, and minimizing the lighting load in an office room in Montreal, Canada. Galapagos utilized a fitness function to determine the goal it needed to solve, with the parametric variables’ relationships defined as outputs (genomes) in this iterative process. The data recorder component in Grasshopper was employed to record the outputs of each generation, saving the data in Microsoft Excel for further analysis. The estimation of energy generation by PV layers involved determining the amount of radiation each layer could absorb. This information was then incorporated into a global formula to estimate the electricity generated by the photovoltaic systems. The dynamic PV facade was implemented on the south face of the office space with an 85% Window Wall Ratio (WWR) and spatial dimensions of 6 m (width), 7 m (depth), and 3 m (height) (Figure 4). The research process is outlined in three phases, as detailed below.

- In the initial phase, the study investigated the dynamic behavior of the dynamic PV facade in controlling daylight uniformity and energy consumption at four different Hours of the Year (HOY). A parametrized prototype was developed, and critical DF and DGP indices for indoor visual comfort were identified through Grasshopper’s optimization capabilities. The suggested glare comfort criteria for DGP included categories such as ‘imperceptible’, ‘perceptible’, ‘disturbing’, and ‘intolerable’. The DF threshold for this analysis was set at 500 Lux. The Honeybee plug-in, integrated with Radiance and Daysim 3, facilitated the analysis of daylight performance and glare probability. An evolutionary solver function called Galapagos, a GA framework within Grasshopper, was utilized to find optimal solutions for dynamic PV facade system positions. Simulations were conducted for 1 P.M. on the 30th of June, 20th of July, 8th of August, and 3rd of September, corresponding to HOY: 4333, 4802, 5175, and 5919.
- The second phase involved determining the optimal positions of PV layers and two artificial lighting control systems to control DF and DGP, aiming to identify configurations that could achieve significant reductions in lighting load. The analysis of lighting load was performed using EnergyPlus 22.2.0 within the Honeybee plug-in. Daily simulations were conducted on the 30th of June, 20th of July, 8th of August, and 3rd of September.
- In the final phase, after identifying the optimum positions of dynamic PV facade layers and an efficient lighting control system, the study assessed the impact of the PV facade system on energy consumption and energy generation. Monthly simulations were conducted for June, July, August, and September, representing the sunniest times of the year in Montreal, and selected using Meteonorm 7 software (global meteorological database for engineers, planners, and education). The assumption in this phase was that each month had an optimum PV layer position, and layers rotated monthly. It is worth noting that, in reality, the layers in the dynamic facade system can rotate on a smaller time scale (sub-hourly) through Lux sensors, actuators, and a Direct Digital Controller to continuously enhance visual comfort and reduce energy consumption.

## 3. Results and Discussion

This section presents the simulation results of the integrated control systems, namely the dynamic PV facade and artificial lighting control. The EnergyPlus Weather File (EPW) for Montreal, along with direct sunlight using the CIE sky, was utilized in this simulation process. The results and related data from all possible generations were categorized into three main sections for discussion:

- DF Analysis and DGP Analysis: This includes an analysis with a threshold of 500 Lux for DF and a threshold of DGP < 0.35 for DGP. The time scale for this analysis spans four different HOYs: 4333, 4802, 5175, and 5919.
- Artificial Lighting Control System Analysis: This section focuses on the analysis of the artificial lighting control system, with a threshold set for achieving the minimum lighting load. The time scale for this analysis covers four specific days.
- Electricity Energy Consumption and Generation: This involves the evaluation of electricity energy consumption in a simulated office room in Montreal and the generation by optimum positions of PV layers in the dynamic facade system. The time scale for this analysis spans four months.

The optimization involved 288 generations for DF and DGP analysis and 3456 generations for lighting load, employing an evolutionary solver known as a GA within Grasshopper. These categories provide a comprehensive overview of the performance and effectiveness of the integrated control systems in various aspects and time scales.

### 3.1. DF and DGP 

**Figure 9:**
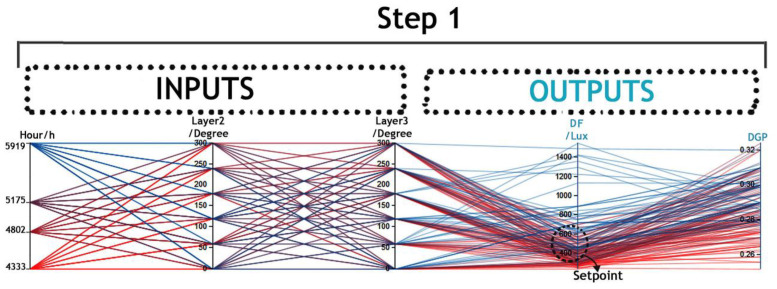
Optimization of Daylight Factor (DF) and Daylight Glare Probability (DGP) using Visual Data Mining Techniques..

In this step, the variations included the rotation of PV layers in six steps and different simulation periods (as shown in Figure 9, step 1). The optimization process generated extensive data and numerous generations, posing the challenge of finding the optimal trade-off between DF and DGP. To address this, data mining techniques were employed to extract actionable information from the optimization data obtained through the GA approach.

A Decision Support System was enhanced using DM, with the online software Design Explorer 2 applied to analyze the multi-objective behavior of the prototype. DE facilitated a better understanding of the results, employing visual data mining techniques to display multivariate datasets in parallel coordinates. This approach transformed the search for relationships among variables into a 2D pattern.

**Figure 10:**
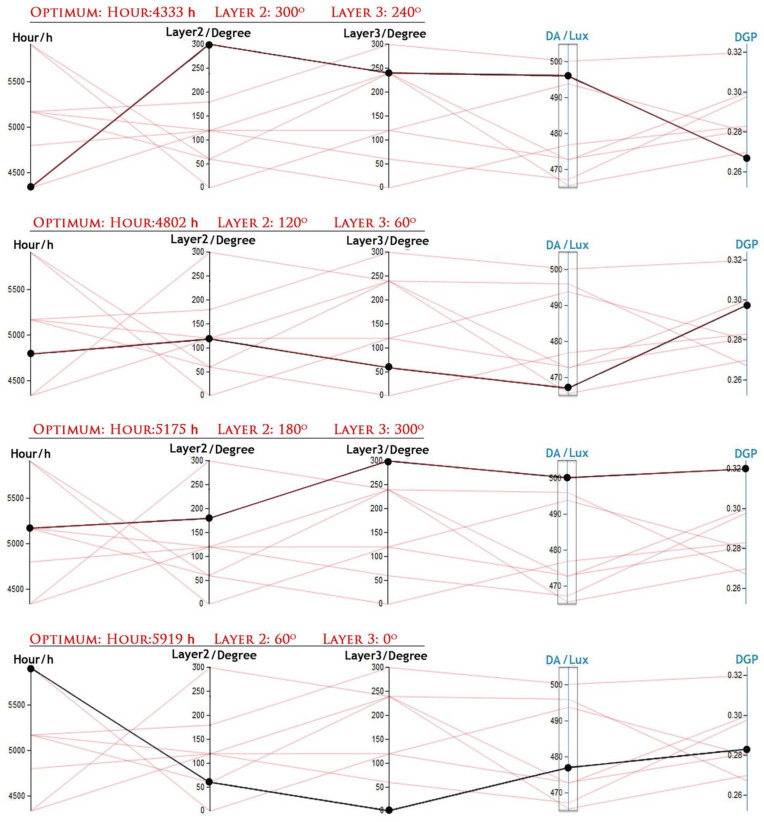
Detecting visual cues to find optimum layers of positions in DF and DGP optimization results.

The results from GA, along with inputs and outputs, were documented in Microsoft Excel spreadsheets. Figure 9 illustrates the visual relationships between multivariate inputs and outputs created by VDM to detect visual cues. In this analysis, a specific domain between 450 and 500 lux (representing acceptable DF) was chosen as a setpoint to identify correlations between DF domains and DGP, as shown in Figure 10.

In Figure 10, the optimal relationships between inputs and outputs to enhance visual comfort during four different HOYs were identified. The outcomes for each simulation period are as follows:

- June (HOY: 4333): Optimal angles for PV layers 2 and 3 were 300° and 240°, respectively. The outputs were 495 lux for DF, representing the average amount of lux over broad surfaces like a whole room, and 0.27 for DGP, falling within the imperceptible glare range.
- July (HOY: 4802): The optimal angle for PV layer 2 was 120°, and for layer 3, it was 60°. The results showed 468 lux for DF and 0.29 for DGP, with the Glare Comfort Range (GCR) categorized as imperceptible glare.
- August (HOY: 5175): An optimal angle of 180° was identified for PV layer 2, and for PV layer 3, 300° was optimal. DF was measured at 500 lux, with DGP at 0.32, falling within the imperceptible glare range.
- September (HOY: 5919): During this simulation period, PV layers 2 and 3 were set at 60° and 0°, respectively. The resulting outputs were DF: 478 lux and DGP: 0.28, with GCR falling within the range of imperceptible glare.

It is crucial to highlight that the dynamic PV facade effectively fulfills the essential building envelope functions concerning air, moisture, and heat transfer, encompassing vital features such as rainscreen protection, water-shedding mechanisms, air and vapor barriers, as well as insulation layers through PV layer 1. PV layer 1, characterized by high transparency, minimally affects daylighting within the space. Its design prioritizes natural ventilation capable of opening during favorable conditions and closing during winter months. However, it is worth noting that while the dynamic facade system enhances natural ventilation, the detailed investigation of its improvement lies beyond the scope of this paper.

The results of Daylight Autonomy (DA), DGP, and GCR calculations in an office room with a dynamic PV facade were compared with a baseline office room without the dynamic PV facade, as presented in Table 4. This comparison aimed to understand the impact of the dynamic PV facade on visual comfort. The table reveals that, in the baseline office room, there is visual discomfort in all four simulation periods, indicated by DA values exceeding 500 lux and DGP values surpassing 0.35. Additionally, the GCR in three HOYs is categorized as intolerable glare.

**Figure 11:**
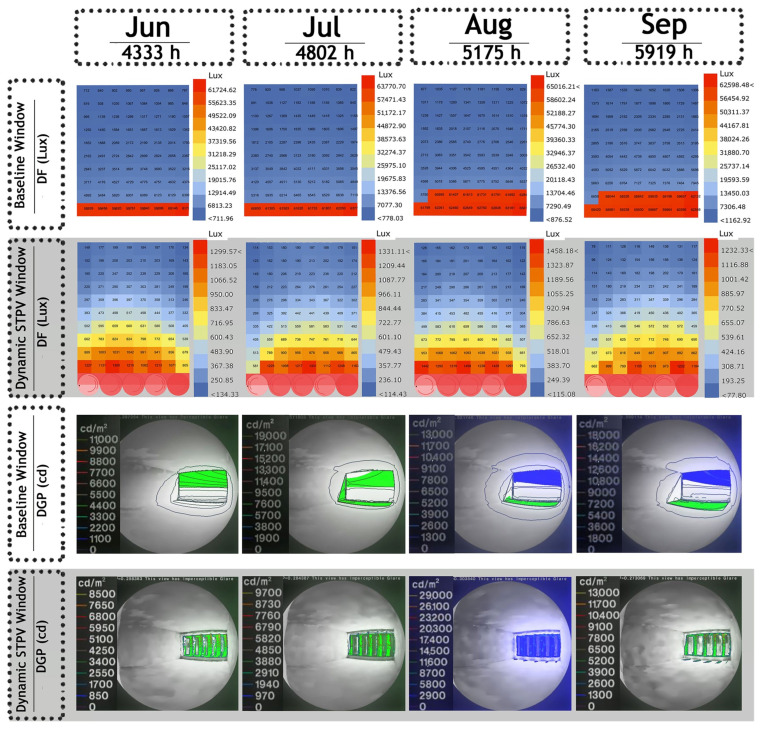
**Above**: (Top View) The ratio of the daylight illumination at points on the work plane in DF analysis. **Below**: (Perspective) The viewpoints chosen in the interior office room space and the radiance picture format in DGP analysis.

Figure 11 presents the grids used for grid-based daylight simulation in DF analysis and the monitor view for image-based simulation in DGP calculation. This comparison is made between the baseline office room and the office room equipped with optimum control systems of dynamic PV facade, as outlined in Table 4. The visual results in Figure 11 demonstrate that the use of PV materials in three layers enables sunlight penetration through diffuse transmission and effectively controls daylight to balance within the standard illuminance range.

Despite the improvement in sunlight penetration, light quality, and uniformity achieved by adopting a dynamic PV system, the results indicate that the standard illuminance level cannot reach deeper into the space in the designated zone. Consequently, the implementation of artificial lighting was deemed necessary to achieve a suitable illuminance domain in all parts of the room.

**Figure 12:**
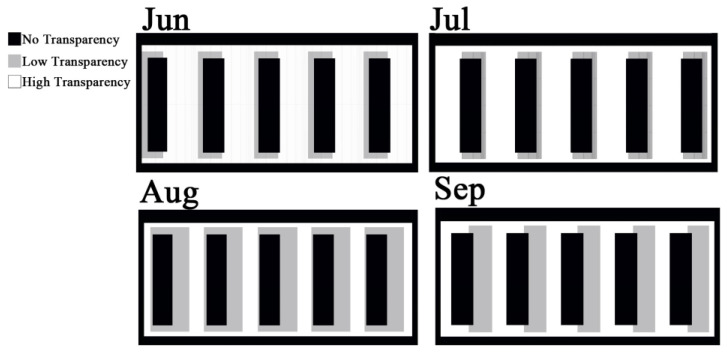
The analysis of the probability of the dynamic PV facade system in optimum angles to have a view outside.

Addressing a common concern regarding BIPV and dynamic facade systems potentially obstructing views outside, Figure 12 illustrates that, with the optimal degree of PV layers, users can still have a view outside. The white space represents areas covered with high-transparency PV, allowing users to see outside, while the black surface consists of PV opaque material with no transparency, and the grey region represents semi-transparent PV.

### 3.2. Lighting Control System

In the subsequent phase following optimization, optimal positions were determined based on predefined set points in terms of DA and daylight DGP for four selected HOYs. This section delves into the evaluation of two distinct artificial lighting control systems: (1) an occupancy sensor system and (2) a dimming system. The optimization inputs encompassed four specific days of the year, the optimal positions of the dynamic PV layers in degrees, and the characteristics of the two lighting control systems.

**Figure 13:**
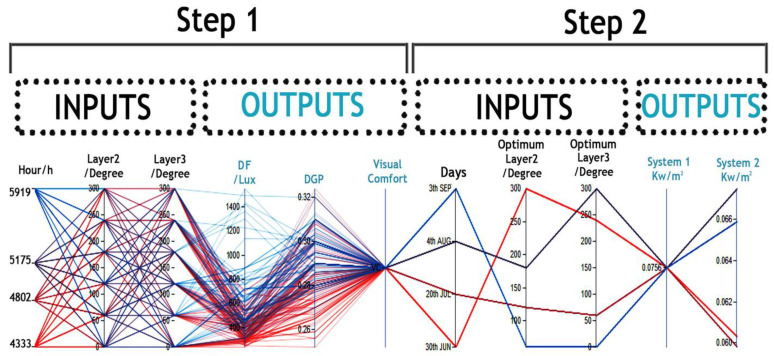
The visual data mining between the results of DF–DGP and lighting control system optimizations.

**Figure 14:**
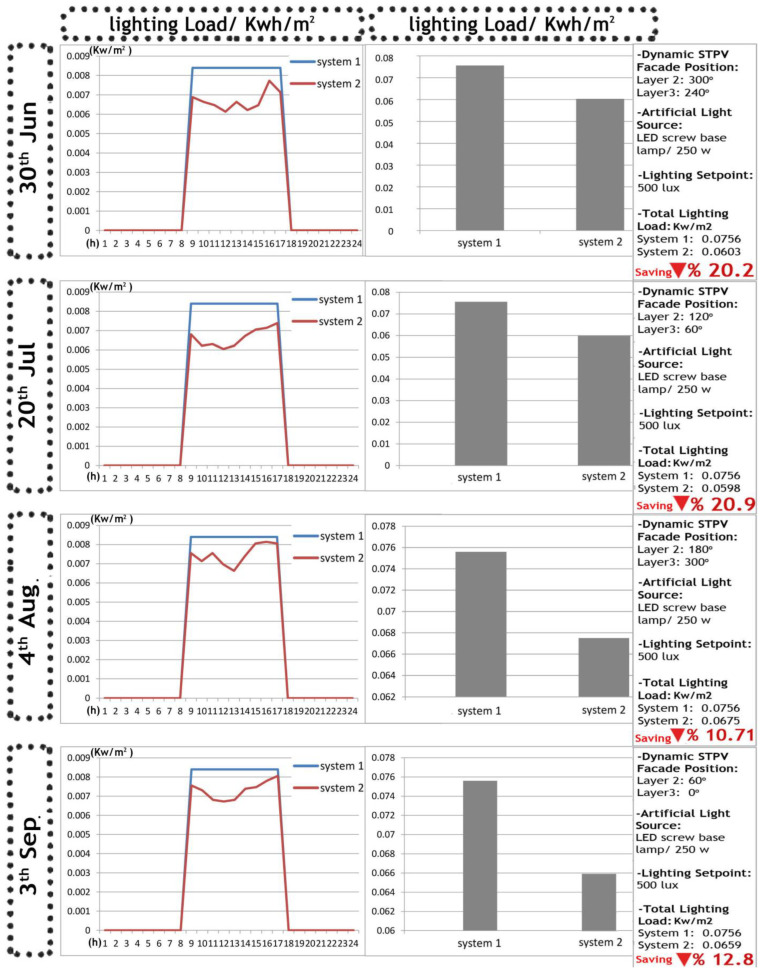
The hourly energy consumption in four simulation days in System 1 (occupancy sensor) and 2 (dimming lighting system).

The VDM results depicted in Figure 13 reveal that the lighting load associated with the occupancy sensor system surpasses that of the dimming system when integrated with the dynamic PV facade system. This outcome arises from the continuous occupation of the office room during working hours, coupled with the improved lighting uniformity facilitated by the dynamic facade system. Furthermore, Figure 14 provides an overview of the hourly energy consumption on the four simulation days for both lighting control systems. Notably, System 2, the dimming system, exhibits a noteworthy energy-saving performance, preserving 20.2% more energy in lighting on 30th June, 20.9% on 20th July, 10.71% on 4th August, and 12.8% on 3rd September. These energy savings contribute significantly to the reduction of peak loads and demand charges during operational hours.

### 3.3. Energy Generation

The energy generation in photovoltaic (PV) layers fluctuated based on their levels of transparency, specific PV properties (refer to Table 1), and the degree of layer orientation. To estimate PV energy generation, we employed Equation (4). Initially, the Daysim engine calculated the hourly radiation each layer could absorb at optimal positions.

**Figure 15:**
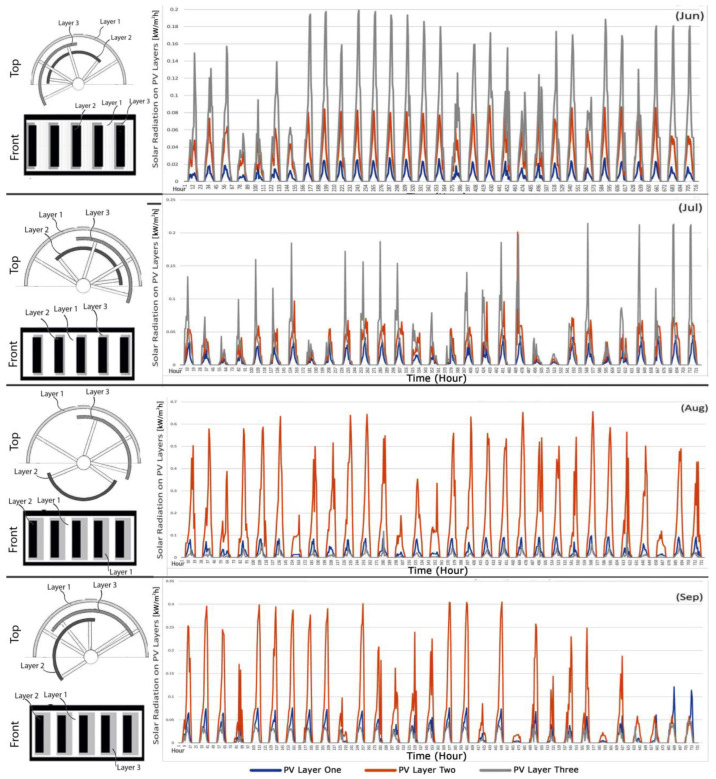
**Left**: The optimum PV layer positions. **Right**: Insolation incident on PV layers in optimum angles found in the last section of this research.

Figure 15 displays the average hourly incident isolation data on PV layers set at optimal angles over a four-month period. The positions of the PV layers were optimized to enhance visual comfort, which means that the layers might not always be oriented to achieve maximum absorption and electricity generation. Table 5 shows the optimal degree of PV layers.

The degree of highly transparent PV layer 1 is zero in all positions in this study because it has minimal effect on daylighting within the space. Its design emphasizes natural ventilation, enabling it to open during favorable weather and close during winter months. However, it is important to note that while the dynamic facade system improves natural ventilation, a thorough examination of these enhancements is outside the scope of this paper. During June and July, PV layer 3 demonstrated higher absorption rates due to its position. Conversely, in August and September, the sun’s angle resulted in PV layer 2 absorbing more radiation. These variations underscore the importance of considering both visual comfort and energy generation efficiency when positioning PV layers.

**Figure 16:**
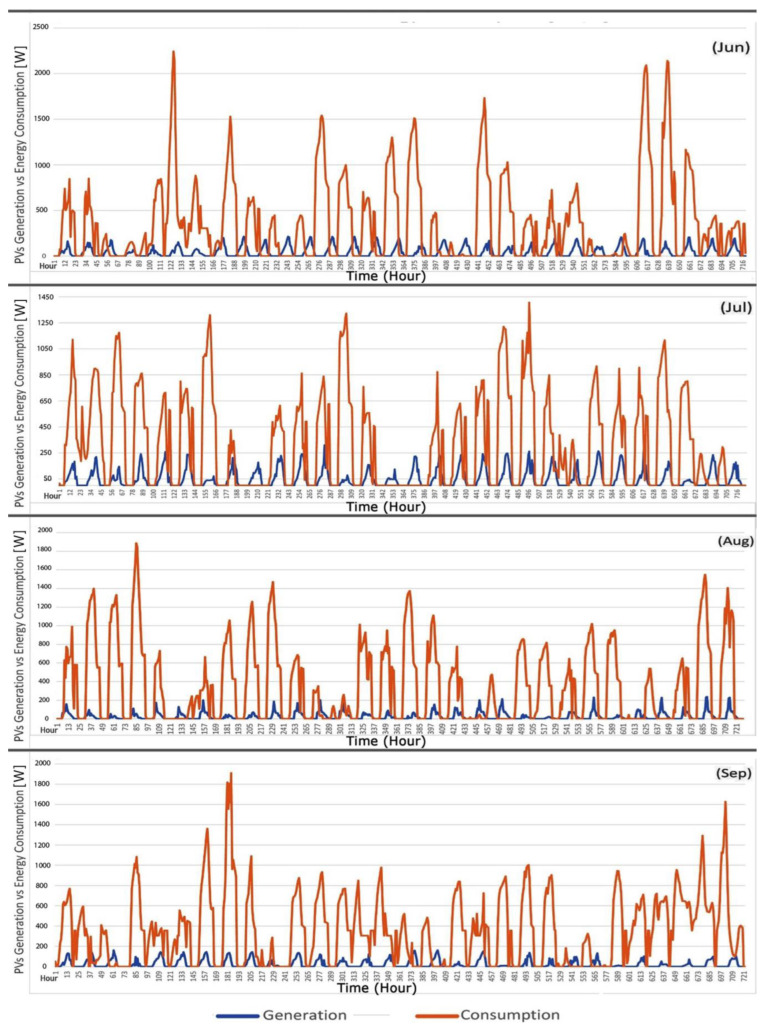
The hourly energy generation and consumption in four months.

The total energy generation of PV layers and the energy consumption of this office room are shown in Figure 16. Typically, the total generation is used immediately by users, and storage is unnecessary. However, having an electric battery for weekends and holidays, when the office is unoccupied, can store electric generation during periods of low solar radiation or reduce peak load during the first hours of working time.

**Figure 17:**
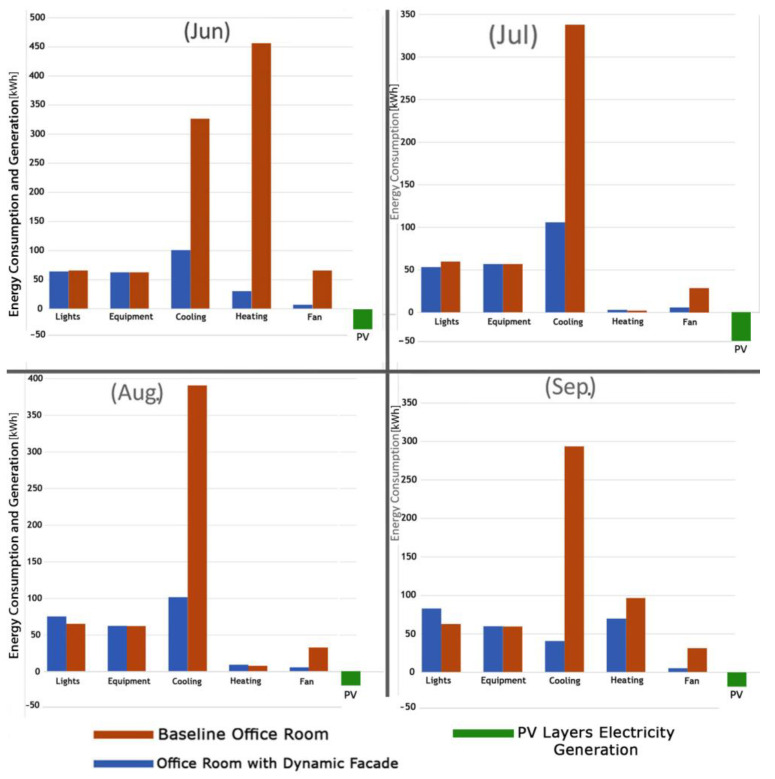
PV energy generation and energy consumption in the baseline office room and the office room with a dynamic PV facade system and control strategy.

Figure 17 compares the energy consumption in the baseline office room and the office room with a dynamic PV facade system for four months. The dynamic PV facade reduces total energy consumption by 73% in June, 54% in July, 54.5% in August, and 52.55% in September. Significant savings are observed in heating and cooling, attributed to the dynamic facade system’s ability to balance and control solar radiation during working hours.

In this context, the average total electricity generation of PV layers in June was 33.21 kWh, representing 12.57% of energy demand. In July, electricity generation was 36.53 kWh, equal to 16.23% of energy demand. For August, electricity generation was 18.82 kWh, accounting for 7.26% of energy demand, and in September, electricity generation was 18.15 kWh, constituting 7% of energy demand. Overall, compared to the baseline office room, the office room with a dynamic PV facade system and control strategy demonstrates energy savings of 76.4% in June, 61.45% in July, 57.8% in August, and 55.88% in September, showcasing the system’s ability to enhance visual comfort, reduce energy consumption, and generate electric energy through PV layers’ rotation and varying transparency levels.

## 4. Conclusions

Dynamic PV facades, inspired by biomimetics, have the potential to significantly improve energy efficiency, visual comfort, and electricity generation in office buildings. By mimicking natural systems that adapt to environmental changes, these facades optimize visual comfort, reduce glare, and minimize energy consumption in glazed office buildings. However, further work is needed to achieve a harmonious balance between these components.

The study shows that dynamic PV facades, demonstrated with a novel design concept, could play a crucial role in future architectural advancements by enhancing energy performance, improving indoor comfort, and contributing to sustainability. As illustrated in the simulation results, optimizing the positioning and transparency of PV layers is essential to maximize this potential. The integration of a dynamic PV facade system allows for precise regulation of daylight and solar radiation, leading to improved visual comfort, reduced energy consumption, and increased electricity generation. The design of the dynamic PV facade also incorporates features such as glare reduction, thermal comfort enhancement, and adaptable transparency levels, making it suitable for various climatic conditions and user needs. The dynamic PV façade significantly reduces energy consumption and enhances visual comfort. It achieves energy savings of 73% in June, 54% in July, 54.5% in August, and 52.55% in September, primarily by controlling solar radiation. Additionally, it generates electricity, covering 7% to 16.23% of energy demand during these months. Overall, the system demonstrates its effectiveness in reducing energy usage and producing electricity, showcasing an average energy savings of up to 76.4% compared to a baseline office room.

This research identifies several key areas for future exploration in dynamic PV facades. These include studying their performance in different climatic regions to assess adaptability and effectiveness, investigating long-term sustainability and practicality, and exploring seamless integration with building automation systems for enhanced efficiency and user experience. Additionally, researching advanced materials for PV layers, applying computational simulations and AI to optimize facade performance, and advancing control systems to improve responsiveness and energy management are crucial. Assessing cost-effectiveness and financial viability to promote broader adoption and evaluating maintenance requirements in large-scale projects are also essential.

In summary, the usefulness of dynamic PV facades lies in their ability to enhance sustainability and efficiency in building operations. They generate electricity, reduce the need for artificial lighting, and improve occupant comfort, thereby promoting innovative and sustainable building design practices rooted in the principles of biomimetics. Further research is vital for developing cost-effective and sustainable dynamic PV facades that meet environmental needs, enhance indoor comfort, and contribute to the overall energy efficiency of buildings.

## Figures and Tables

**Table 4:** The results of DA, DGP, and GCR with and without dynamic facade on an office room.

	Jun. HOY: 4333	July. HOY: 4802	Aug. HOY: 5175	Sep. HOY: 5919
Baseline (Lux)	8286	8691	13,876	14,099
Baseline with Dynamic Facade (Lux)	495	468	500	478
Baseline (DGP)	0.39	0.51	0.52	0.58
Baseline with Dynamic Facade (Lux)	0.27	0.29	0.32	0.28
Baseline (GCR)	Perceptible Glare	Intolerable Glare	Intolerable Glare	Intolerable Glare
Baseline with Dynamic Facade (Lux)	Imperceptible Glare	Imperceptible Glare	Imperceptible Glare	Imperceptible Glare

**Table 5:** The optimal degree of PV layer orientation.

Month	Optimal Angle, PV Layer 1 (Degree)	Optimal Angle, PV Layer 2 (Degree)	Optimal Angle, PV Layer 3 (Degree)
June	0	300	240
July	0	120	60
August	0	180	300
September	0	60	0

## Data Availability

Data are contained within the article.
